# Children’s exposure to herbicides in Jalisco, Mexico—a public health concern perspective

**DOI:** 10.3389/fpubh.2025.1659996

**Published:** 2025-10-15

**Authors:** Felipe de Jesús Lozano-Kasten, Alejandro Aarón Peregrina-Lucano, Josefina Fausto-Guerra, Norma Guadalupe Ruiz Velazco-Sandoval, Daniel Flores-Rodríguez, Horacio Guzmán-Torres

**Affiliations:** ^1^Departamento de Salud Pública, Centro Universitario de Ciencias de la Salud, Universidad de Guadalajara, Guadalajara, Mexico; ^2^Departamento de Farmacobiología, Centro Universitario de Ciencias Exactas e Ingenierías, Universidad de Guadalajara, Guadalajara, Mexico; ^3^Departamento de Enfermería para la Atención, Desarrollo y Preservación de la Salud Comunitaria, Centro Universitario de Ciencias de la Salud, Universidad de Guadalajara, Guadalajara, Mexico; ^4^Hospital General Regional No. 45, Salud en el Trabajo, Instituto Mexicano del Seguro Social, Guadalajara, Mexico

**Keywords:** children’s health, herbicides, exposure, public health, environmental health

## Abstract

This perspective article examines public health concerns related to herbicide exposure among children living in an agricultural and fishing village on the shores of Lake Chapala in Jalisco, Mexico. The discussion draws on published research comprising four pesticide exposure assessments conducted on children aged 0–14 years between 2016 and 2018. These assessments involved the collection of first-morning urine samples, which were analyzed using high-performance liquid chromatography coupled with tandem mass spectrometry (HPLC-MS/MS). The findings are as follows: November 2016 (*n* = 347; 24.2% positive; 7 herbicides detected), October 2017 (*n* = 187; 86.6% positive; 6 herbicides detected), May 2018 (*n* = 347; 52.2% positive; 4 herbicides detected), and October 2018 (*n* = 347; 12% positive; 4 herbicides detected). A total of three herbicides—glyphosate, 2,4-D (2,4-dichlorophenoxyacetic acid), and molinate—were detected in each of the four assessments. These results may represent the first evidence of persistent herbicide exposure in children in the Lake Chapala region. Further epidemiological studies are required to deepen the understanding of these findings.

## Introduction

Children have been unintentionally exposed to diverse xenobiotics that proliferated after World War II. In this context, pesticides were developed as chemical mixtures designed to control or eliminate undesirable forms of life, such as animals or plants. Herbicide use became widespread in pursuit of increased food production. However, studies on long-term exposure to these potential pollutants and their associated health effects are scarce and are primarily focus on acute health risks. Biomonitoring of exposed populations establishes links to diseases suspected of being pesticide-related, using non-invasive biomarkers such as urine. This knowledge is essential, as pesticides constitute a cornerstone of global agricultural technology, are also available for domestic use, and consequently expose children to residues through the consumption of fruits and vegetables (1–4).

Herbicides are applied to 85–100% of crops, including corn, soybeans, oats, cotton, and rice, in conjunction with the use of Genetically Modified Organisms (GMOs). GMO crops are engineered to resist pests, pesticides, and drought, offering improved production performance and consumer-preferred characteristics in fruits and vegetables. However, no scientific consensus exists regarding the health effects of consuming GMO, and health risks are primarily associated with the persistence of pesticide residues due to increased herbicide use. This practice is widespread in developing countries, driven by economic savings in agricultural activities (5–8).

As previously noted, children’s health is influenced by multiple factors stemming from human activities in the environment (2). These include biodiversity loss, the depletion of non-renewable resources, and the proliferation of resistant microorganisms, such as viruses and bacteria, which are recognized risk factors contributing to diverse and often unpredictable effects. The interactions among the environment, economy, and community health have an interdependent relationship, known as the ecosystemic perspective (9, 10).

## Geographical and community context

**Figure 1:**
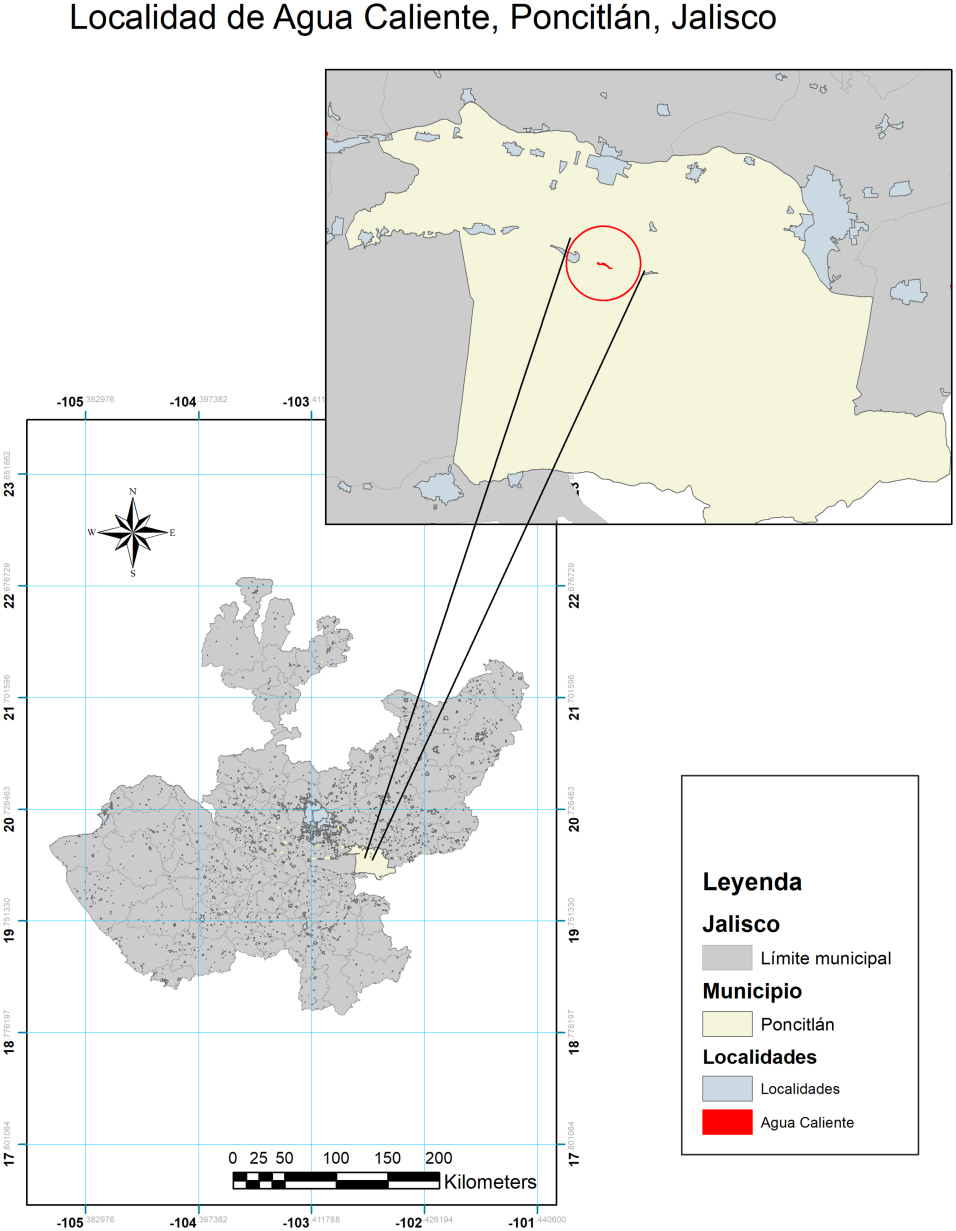
Agua Caliente, Poncitlán, Jalisco, México, Geographic Coordinates 20.31173, −102.926021. Source: Map author: Horacio Guzmán-Torres. Map Maker: Melva Guadalupe Herrera-Godina. Software Argis version 10.6 (33).

In this perspective article, the aim was to examine public health concerns related to herbicide exposure among children living in an agricultural and fishing village on the shores of Lake Chapala in Jalisco, Mexico (Figure 1).

The authors selected Agua Caliente as the study area following a 2016 alert from local health authorities regarding an unusual increase in the prevalence of kidney disease among children in this region. Consequently, the Chapala Project was established at the University of Guadalajara to investigate other potential health risk factors. Initial findings revealed that the population (N = 998, with 550 individuals under 15 years old) lived in conditions of social and economic hardship (11). Their primary livelihoods included farming (37.9%), construction work (29.3%), and manual labor (7.2%), supplemented by fishing primarily for subsistence. Common crops, such as seasonal maize, beans, and chayote (*Sechium edule*), are irrigated with water from Lake Chapala. The average weekly household income was approximately 52.63 USD. Regarding kidney health, the study reported a prevalence of non-traditional Chronic Kidney Disease (CKDnt) characterized by albuminuria, with rates 3–5 times higher than those documented in international literature. Among the 394 children (0–17 years old) assessed, 68.1% were classified as having stage 3a or 3b CKDnt according to the Kidney Disease: Improving Global Outcomes (KDIGO) classification (12).

## The herbicide exposure in Agua Caliente

The findings presented are derived from the published study “Childhood Exposure to Agricultural Chemicals in Jalisco: A Crisis of Knowledge, Awareness, and Health” by Vega-Fregoso et al. (13). This study encompasses four pesticide exposure assessments conducted on children aged 0–14 years between 2016 and 2018. The assessments involved collecting first-morning urine samples, which were analyzed using high-performance liquid chromatography coupled with tandem mass spectrometry (HPLC-MS/MS). The results are as follows: November 2016 (*n* = 347; 24.2% positive; 7 herbicides detected), October 2017 (*n* = 187; 86.6% positive; 6 herbicides detected), May 2018 (*n* = 347; 52.2% positive; 4 herbicides detected), and October 2018 (*n* = 347; 12% positive; 4 herbicides detected) (13). These pesticide detection results are summarized in Table 1.

**Table 1:** Herbicide exposure detection in children (<14-year old) residing in Agua Caliente, Jalisco, Mexico, 2016–2018.

Pesticides detected	Nov 2016 *n* = 347 (24.2%)*	Oct 2017 *n* = 187 (86.6%)*	May 2018 *n* = 347 (52.2%)*	Oct 2018 *n* = 347 (12%)*
Acetochlor	LOD	+	LOD	LOD
Atrazine	+	+	LOD	LOD
Glyphosate	+	+	+	+
Metoxuron	+	+	LOD	LOD
2,4-D 2,4-(Dichlorophenoxyacetic acid)	+	+	+	+
Molinate	+	+	+	+
Picloram	+	LOD	+	+
Total of herbicides detected	7	6	4	4

Data from “Childhood Exposure to Agricultural Chemicals in Jalisco: A Crisis of Knowledge, Awareness, and Health” (13).

+ detection of pesticides.

*Proportion of children with at least one detectable pesticide.

LOD (limit of detection 0.01 to 1,000 ng/mL). The lowest quantity or concentration of a component that can be reliably detected.

The HPLC-MS/MS method is described in Sierra-Díaz et al. (11).

## Discussion

It is important to note the limitations of the 2016–2018 pesticide exposure assessment conducted by Vega-Fregoso et al. (13), which serves as the basis of this perspective article. The study provides only qualitative data, lacking quantitative information on herbicide concentrations. Consequently, it is not possible to estimate exposure levels, compare them to safety thresholds, or conduct statistical analyses.

In the 2016–2018 Agua Caliente study, the detection of three herbicides—glyphosate, 2,4-D (2,4-dichlorophenoxyacetic acid), and molinate—in each of the four assessments provides evidence of persistent herbicide exposure among children aged 0–14 years. Building on these findings, a cross-sectional study conducted in 2019 analyzed pesticide levels in first-morning urine samples from children residing in two farming communities: Agua Caliente (*n* = 192, aged 5–15 years) and Ahuacapan (*n* = 89, aged 5–13 years). Positive levels of at least two pesticides were detected in 100% of the study participants, with malathion, methoxuron, glyphosate, dimethoate, and acetochlor present in 70% of children in both communities. Ahuacapan, located approximately 112 miles from Agua Caliente, has a population of 950 inhabitants. The authors noted that differences between the two communities are linked to the nature of children’s involvement in pesticide handling. In Agua Caliente, children often assist their relatives with crop management activities, including pesticide application, primarily for subsistence farming. In contrast, children in Ahuacapan are frequently employed as seasonal agricultural workers (11).

A cross-sectional study conducted in Guadalajara by Gomez-Ruiz et al. (14), analyzed urine samples from 280 neonates at Hospital Civil Guadalajara to detect nine herbicides using high-performance liquid chromatography coupled with tandem mass spectrometry (HPLC-MS/MS). Three herbicides were identified: molinate [(67 cases, 23.9%; mean 408 ng/mL, standard deviation SD 229.3)], 2,4-D (64 cases, 22.8%; mean 38 ng/mL, SD 5.6), and glufosinate (34 cases, 12.1%; mean 575 ng/mL, SD 385.2). The authors identified the maternal pathway as the primary route of exposure to herbicides. Notably, the mothers reported no involvement in herbicide application activities (14).

Regarding environmental findings in regional studies, Rodríguez-Aguilar et al. (15) identified 11 pesticides in fish muscle samples from the Ayuquila–Armería River area, known for its cattle and agricultural activities, located approximately 100 miles from Agua Caliente. Among the 11 analytes detected, *λ*-cyhalothrin, ametrine, and malathion exhibited the highest concentrations, while carbendazim, malathion, and glyphosate showed the highest frequency of detection (>70%) (15). In the same region, the authors identified pesticide residues, including imazalil, picloram, malathion, *λ*-cyhalothrin, emamectin, and methomyl, in the feces of otters (16). Additionally, a cohort study conducted in 2015, 2016, and 2017 detected 17 pesticides in water samples from 13 different sites along the river, with 2,4-D, malathion, glyphosate, and molinate present in at least one of the six sampling events (17).

In Agua Caliente, maize is the primary food source for local communities, with harvesting practices transitioning over the past 40 years from traditional, pesticide-free methods to industrial agriculture reliant on chemical inputs. The region experiences only two seasons—a rainy season and a dry season—which influence the timing of herbicide, insecticide, and fertilizer applications, as well as seeding, harvesting, threshing, and corn storage. This practice is characteristic of seasonal agriculture. Children in the Mexican state of Jalisco are involved in agricultural activities in a similar manner, but they face different challenges. They work as seasonal migrants or as support personnel to help relatives (11). In the first case, migrant children’s labor in agriculture represents income for their families, replacing adult family members and enabling new opportunities, but it also creates difficulties in accessing education. Seasonal agricultural migration is typically informal, but it often leads to unjust and abusive situations (18).

The presence of herbicides in children’s bodies poses a significant public health challenge, particularly due to potential long-term health effects. One contributing factor is the lack of surveillance in industrial-agricultural environments, as only agro-industries are required to comply with environmental and food safety regulations. This limited oversight hinders the prediction, toxicity assessment, and risk management of agrochemical exposure, which can occur through various sources, including food, water, domestic use, yards, and schools (3). Surveillance of herbicide exposure is further complicated by the fact that these chemicals can be applied by anyone, including individuals without formal training in their use. In Mexico, no license or identification is required to purchase agrochemicals, allowing unrestricted access. This lack of regulation may lead to improper handling, application, and disposal of pesticide containers, inadequate use of personal protective equipment, the use of obsolete or banned pesticides, and an increased risk of contaminating drinking water and soil (19).

In the 2016–2018 pesticide exposure assessment conducted by Vega-Fregoso et al. (13), a total of seven herbicides were identified: acetochlor, atrazine, glyphosate, metoxuron, 2,4-D, molinate, and picloram. All of them have been widely used, and none of them are beneficial for children’s health. In regard of health effects, the acetochlor exposure have been linked with cytotoxicity effects on wildlife and human (20), atrazine adverse health effects due to diverse exposure times and periods impact many different systems in human body but mainly neuroendocrine toxicity (21), glyphosate-based herbicides (GBH) are linked to an increased risk to non-Hodking lymphoma (NHL) according to meta-analysis of human epidemiological studies (22), in case of metoxuron, results in literature remains limited to 38 aimed to ecotoxicity mainly and it is described as long-term hazard with high toxicity for aquatic environments but human exposure and effects are not met (23), the herbicide 2,4-D (2,4-dichlorophenoxyacetic acid) is a component of Agent Orange, a military defoliant (24), remarkably, this is one of the most commonly used selective herbicides in the world and authors report evidence of association between NHL and exposure to 2,4-D (25). The information on molinate exposure and effects is scarce, but it belongs to the category of thiocarbamate herbicides, which are not approved for use in the United States or European Union; however, human exposure to carbamates and thiocarbamates is associated with cholinergic syndrome (26) and neurotoxicity in the peripheral nervous system (27). Picloram is an organochlorine persistent herbicide; however, no data are available from human studies, and limited evidence is available from studies in experimental animals using technical grades. It is not classifiable as a human carcinogen, and hepatic critical effect system has been reported (28).

In Mexico, glyphosate has been at the center of controversy, prompting several attempts to restrict its use due to environmental and public health concerns. In February 2023, the federal government issued restrictions on glyphosate and genetically modified (GMO) maize. However, these measures were revoked in February 2025 following a NATO panel agreement to avoid international economic sanctions (29).

Although long-term studies on human pesticide exposure remain limited, environmental susceptibility is a critical factor that may influence disease development. Relevant factors include exposure to chemicals, diet, physical activity, alcohol and tobacco consumption, genetic predisposition, and their interactions with the environment, as well as pre-existing health conditions (30).

In summary, the herbicides glyphosate, molinate, and 2,4-D were detected in each of the four assessments conducted during the 2016–2018 project. Glyphosate, classified by the World Health Organization’s International Agency for Research on Cancer (IARC) in 2015 as “probably carcinogenic to humans” (Group 2A), has been subject to ongoing debate (31). This classification was based on limited evidence of carcinogenicity in humans from real-world exposures and sufficient evidence from experimental animal studies using pure glyphosate. However, assessments by the European Union and the Food and Agriculture Organization of the United Nations (FAO) have not corroborated these findings, leading to a lack of consensus that delays the establishment of unified safety standards to protect environmental and public health (32).

Exposure to glyphosate in both animals and humans has been associated with a range of neurotoxic effects, including disruptions in neurotransmission, induction of oxidative stress, neuroinflammation, and mitochondrial dysfunction. These processes can lead to neuronal death through mechanisms such as autophagy, necrosis, or apoptosis, potentially contributing to behavioral and motor disorders. Researchers note that these effects vary widely depending on dosage but generally occur at levels below the regulatory limits set by agencies, such as the Environmental Protection Agency’s (EPA) Classification IV, which denotes the least toxic category (non-toxic and non-irritating) (32).

This perspective article highlights the importance of conducting longitudinal studies on pesticide exposure that take into account environmental factors and their potential interactions to identify individuals at higher risk of related health outcomes, as a significant portion of Mexico’s agricultural land relies on pesticide-based practices.

## Data Availability

The original contributions presented in the study are included in the article/supplementary material, further inquiries can be directed to the corresponding author.
